# Feasibility of cystatin C as a biomarker for AECOPD severity: a cross-sectional study integrating CAT score, mMRC grade, and GOLD stage

**DOI:** 10.3389/fmed.2026.1804455

**Published:** 2026-04-09

**Authors:** Haoran Ma, Mengwen Yan, Jun Liu, Chen Zhu

**Affiliations:** 1 Department of Respiratory and Critical Care Medicine, Fuyang Women's and Children's Hospital, Fuyang, Anhui, China; 2 Department of Ophthalmology, Fuyang Women's and Children's Hospital, Fuyang, Anhui, China; 3 Department of Obstetrics and Gynecology, Fuyang Women's and Children's Hospital, Fuyang, Anhui, China

**Keywords:** acute exacerbations of chronic obstructive pulmonary disease, biomarker, CAT score, cystatin C, GOLD staging, mMRC grading

## Abstract

**Background:**

Chronic obstructive pulmonary disease (COPD) exacerbations require accurate severity assessment for optimal management.

**Objective:**

This study investigated serum cystatin C as a potential biomarker for evaluating acute exacerbations of COPD (AECOPD) severity and its correlation with established clinical assessment tools.

**Methods:**

A cross-sectional study enrolled 389 consecutive AECOPD patients hospitalized from 01 January 2024 to 31 December 2025. Patient demographics, laboratory parameters, arterial blood gases, CAT scores, mMRC grading, and GOLD staging were collected. Statistical analyses included Spearman correlation, ANOVA, multiple regression, ROC curve analysis, and restricted cubic spline modeling.

**Results:**

Patients averaged 68.71 ± 0.8 years with 71.7% male pre-dominance. Cystatin C levels progressively increased across CAT score severity groups: mild (< 10 points) 1.080 ± 0.32 mg/L, moderate (10–20) 1.380 ± 0.41 mg/L, severe (21–30) 1.720 ± 0.52 mg/L, and very severe (>30) 2.150 ± 0.68 mg/L (*F* = 78.42, *P* < 0.001). Strong positive correlations existed between cystatin C and CAT scores (*rs* = 0.687), mMRC grading (*rs* = 0.612), and GOLD staging (*rs* = 0.534, all *P* < 0.001). Multiple regression confirmed cystatin C as an independent CAT score predictor (β = 5.89, 95%CI: 4.72–7.06, *P* < 0.001). For severe AECOPD prediction (CAT ≥ 21), cystatin C demonstrated good diagnostic performance with AUC 0.847 (95%CI: 0.807–0.887), optimal cutoff 1.52 mg/L, sensitivity 81.5%, and specificity 78.2%. Restricted cubic spline analysis revealed a significant non-linear dose-response relationship (*P* = 0.023).

**Conclusion:**

Serum cystatin C strongly correlates with AECOPD severity across multiple assessment scales and demonstrates good diagnostic accuracy, supporting its potential as a reliable biomarker for clinical severity evaluation in COPD exacerbations.

## 1 Introduction

Chronic obstructive pulmonary disease (COPD) is a chronic respiratory disorder characterized by persistent airflow limitation and represents the third leading cause of death globally (1). Acute exacerbations of COPD (AECOPD) are defined as clinical events involving acute worsening of respiratory symptoms that require additional treatment, constituting a major factor contributing to increased hospitalization rates and mortality among COPD patients (2). Research demonstrates that each acute exacerbation accelerates lung function decline, significantly impacting patient quality of life and prognosis (3).

Currently, AECOPD severity assessment primarily relies on clinical symptoms, pulmonary function testing, and scoring scales. The COPD Assessment Test (CAT) is a simple, reliable health status evaluation tool that reflects patient symptom burden (4). The modified Medical Research Council (mMRC) dyspnea scale is used to assess the degree of breathlessness (5). The Global Initiative for Chronic Obstructive Lung Disease (GOLD) staging system evaluates airflow limitation severity based on pulmonary function assessment (6). However, these evaluation methods have limitations including high subjectivity and dependence on patient cooperation. Therefore, identifying objective, reliable biomarkers for AECOPD severity assessment is of paramount importance.

Cystatin C (CysC) is a low molecular weight protein produced at a constant rate by all nucleated cells and is primarily eliminated through glomerular filtration (7). Compared to serum creatinine, cystatin C is not influenced by age, sex, muscle mass, or dietary factors, making it an ideal marker for assessing glomerular filtration rate (8). Recent studies have revealed that cystatin C not only reflects renal function status but is also closely associated with inflammatory responses, oxidative stress, and cardiovascular diseases (9–11). In the COPD field, studies have reported correlations between cystatin C levels and disease severity and prognosis (12, 13), although systematic research on cystatin C in AECOPD severity assessment remains limited.

While the role of cystatin C in COPD and inflammation has been initially explored, its potential as a biomarker for assessing disease severity and its specific diagnostic efficacy in hospitalized patients with acute exacerbations of COPD (AECOPD), particularly regarding the establishment of clinically guiding diagnostic cut-off values, still lack support from large-scale, high-quality research data. Existing studies often focus on stable COPD or fail to provide clear clinical application thresholds. Therefore, this study aims to bridge this gap by comprehensively evaluating the correlation between serum cystatin C and various AECOPD severity indicators through an in-depth analysis of a large cohort of hospitalized AECOPD patients. Furthermore, it seeks to first investigate its feasibility as a diagnostic marker for severe AECOPD and determine its optimal diagnostic cut-off value, with the aim of providing an objective and effective risk assessment tool for clinical practice.

## 2 Materials and methods

### 2.1 Study design

This study was a single-center, cross-sectional observational study. The research protocol was approved by the Medical Ethics Committee of our institution (approval number: 2021-KY-089), and written informed consent was obtained from all enrolled patients. The study was conducted in strict accordance with the Declaration of Helsinki and the strengthening the reporting of observational studies in Epidemiology (STROBE) statement.

### 2.2 Study subjects

Inclusion criteria: consecutive enrollment of AECOPD patients hospitalized in the Department of Respiratory and Critical Care Medicine at our institution from 01 January 2024 to 31 December 2025. The inclusion criteria were as follows: (1) Age ≥40 years; (2) Meeting the COPD diagnostic criteria according to GOLD 2023 guidelines: post-bronchodilator FEV_1_/FVC < 0.70 (6); (3) Meeting AECOPD diagnostic criteria: worsening dyspnea, increased sputum volume and/or purulent sputum, with symptom changes exceeding daily variation and requiring modification of treatment regimen (14); (4) Completion of all examinations and assessments within 48 h of admission; (5) Written informed consent obtained from patients or their legal guardians. Exclusion criteria: (1) concurrent respiratory diseases including bronchial asthma, bronchiectasis, pulmonary interstitial fibrosis, pulmonary embolism, or pneumothorax; (2) Acute coronary syndrome, acute heart failure, or severe cardiac arrhythmias; (3) Acute cerebrovascular disease; (4) Severe hepatic dysfunction (Child-Pugh Class C); (5) End-stage renal disease (eGFR < 15 ml/min/1.73 m^2^) or dialysis treatment; (6) Active malignancy or hematologic disorders; (7) Autoimmune diseases or use of immunosuppressive agents within the preceding 3 months; (8) Uncontrolled hyperthyroidism or hypothyroidism; (9) Pregnant or lactating women; (10) Inability to cooperate with CAT scoring, mMRC grading, or pulmonary function testing.

### 2.3 Data collection

The following data were collected by uniformly trained research personnel: demographic information: gender and age. Physical examination: height, weight, body mass index (BMI), systolic blood pressure (SBP), and diastolic blood pressure (DBP). Lifestyle factors: smoking history (defined as cumulative smoking ≥100 cigarettes) and alcohol consumption history (defined as alcohol intake ≥1 time per week on average in the past year). Medical history: diabetes mellitus (based on previous diagnosis or fasting plasma glucose ≥7.0 mmol/L or random blood glucose ≥11.1 mmol/L) and hypertension (based on previous diagnosis or systolic blood pressure ≥140 mmHg and/or diastolic blood pressure ≥90 mmHg). Blood Sample Collection: all patients underwent fasting venipuncture (8–12 h fasting) on the morning following admission. A total of 10 ml of cubital venous blood was collected and distributed into EDTA-K_2_ anticoagulant tubes, sodium citrate anticoagulant tubes, and serum separator tubes. Samples were analyzed within 2 h of collection or centrifuged and stored at −80 °C. Laboratory Parameters: (1) Complete blood count: neutrophil count (NEU, × 10^9^/L); monocyte count (MONO, × 10^9^/L); red blood cell count (RBC, × 10^1^^2^/L); hemoglobin (Hb, g/L); absolute lymphocyte count (LYM, × 10^9^/L); platelet count (PLT, × 10^9^/L). (2) Coagulation function: D-dimer (mg/L). (3) Biochemical markers: fasting plasma glucose (FPG, mmol/L); creatinine (Cr, μmol/L); blood urea nitrogen (BUN, mmol/L); uric acid (UA, μmol/L); albumin (ALB, g/L); C-reactive protein (CRP, mg/L). (4) Cystatin C. arterial blood gas analysis: radial arterial blood (1 ml) was collected from all patients at admission (before oxygen therapy or 30 min after discontinuing oxygen supplementation). Parameters included: pH value; partial pressure of carbon dioxide (PaCO_2_, mmHg); partial pressure of oxygen (PaO_2_, mmHg); base excess (BE, mmol/L); buffer base (BB, mmol/L); lactate (Lac, mmol/L); bicarbonate concentration (${\text{HCO}}_{3}^{-}$, mmol/L).

### 2.4 Assessment of disease severity

CAT score assessment: the validated Chinese version of the COPD Assessment Test questionnaire was employed, encompassing eight domains: cough, sputum production, chest tightness, dyspnea when climbing hills/stairs, limitation in home activities, confidence in leaving home, sleep quality, and energy levels. Each item is scored from 0–5 points, with a total score ranging from 0–40 points. Based on scores, patients were categorized as follows: mild symptoms group (< 10 points), moderate symptoms group (10–20 points), severe symptoms group (21–30 points), and very severe symptoms group (>30 points) (4). mMRC Grading: the modified Medical Research Council dyspnea scale was used to assess the degree of breathlessness, with grades ranging from 0–4 (5): grade 0: breathlessness only during strenuous exercise; Grade 1: short of breath when hurrying on level ground or walking up a slight incline; Grade 2: walks slower than people of the same age on level ground due to breathlessness, or has to stop for breath when walking at own pace; Grade 3: stops for breath after walking approximately 100 meters or after a few minutes on level ground; Grade 4: too breathless to leave the house or breathless when dressing or undressing. GOLD Staging: pulmonary function testing was performed after clinical stabilization (typically before discharge or on hospital days 5–7) using the German Jaeger MasterScreen PFT system. Staging was based on post-bronchodilator FEV_1_ as a percentage of predicted values (6): GOLD Stage 1 (mild): FEV_1_ ≥ 80% predicted; GOLD Stage 2 (moderate): 50% ≤ FEV_1_ < 80% predicted; GOLD Stage 3 (severe): 30% ≤ FEV_1_ < 50% predicted; GOLD Stage 4 (very severe): FEV_1_ < 30% predicted. Comprehensive AECOPD severity classification: a comprehensive assessment combining clinical manifestations, laboratory findings, and arterial blood gas analysis was conducted: mild: CAT score < 10 points, no respiratory failure, no hospitalization required; Moderate: CAT score 10–20 points, or PaO_2_ < 60 mmHg without CO_2_ retention; Severe: CAT score ≥21 points, or concurrent respiratory failure (PaO_2_ < 60 mmHg with PaCO_2_ >50 mmHg), or requiring mechanical ventilation or ICU treatment.

### 2.5 Statistical methods

Statistical analyses were performed using SPSS 26.0, *R* 4.2.1, and MedCalc 20.0 software. Continuous variables were first tested for normality using the Shapiro–Wilk test and homogeneity of variance using Levene's test. Normally distributed continuous variables are presented as mean ± standard deviation ($\bar{\text{x}}$ ± s), while non-normally distributed variables are expressed as median (interquartile range) [M (Q_1_, Q_3_)]. Categorical variables are presented as frequency [percentage; *n* (%)].

For comparisons between two groups, independent samples *t*-test was used for normally distributed data with equal variances, otherwise the Mann–Whitney *U*-test was applied. For multiple group comparisons, one-way ANOVA was used for normally distributed data with equal variances, with *post hoc* comparisons performed using LSD-*t*-test or Bonferroni correction; otherwise, the Kruskal–Wallis *H*-test was employed with Dunn–Bonferroni method for pairwise comparisons. Categorical variables were compared using χ^2^ test or Fisher's exact test. Trend analysis was conducted using linear trend χ^2^ test or Jonckheere–Terpstra trend test.

Bivariate correlations were assessed using Pearson correlation analysis for normally distributed variables and Spearman rank correlation analysis for non-normally distributed or ordinal variables. Partial correlation analysis was performed to examine correlations after controlling for confounding factors. Correlation coefficients were interpreted as follows: |*r*| < 0.3 indicated weak correlation, 0.3 ≤ |*r*| < 0.5 moderate correlation, 0.5 ≤ |*r*| < 0.7 strong correlation, and |*r*| ≥ 0.7 very strong correlation.

Multiple linear regression analysis was conducted with CAT score as the dependent variable, incorporating variables with *P* < 0.05 from univariate analysis into stepwise regression models after testing for multicollinearity (VIF < 5 considered acceptable). Binary logistic regression was performed with severe AECOPD (CAT ≥ 21 points) as the dependent variable to analyze the independent predictive effect of cystatin C. Three models were constructed: model 1 (univariate analysis), model 2 (adjusted for demographic characteristics including age, gender, and BMI), and model 3 (further adjusted for clinical indicators including smoking history, diabetes, hypertension, CRP, and creatinine). Odds ratios (OR) with 95% confidence intervals (CI) were calculated, and model goodness-of-fit was evaluated using the Hosmer–Lemeshow test.

ROC curve analysis was performed to evaluate the predictive performance of cystatin C for different severities of AECOPD, with calculation of area under the curve (AUC) and 95% CI. Optimal cutoff values were determined using the Youden Index (Youden Index = Sensitivity + Specificity – 1), and sensitivity, specificity, positive predictive value (PPV), negative predictive value (NPV), positive likelihood ratio (+LR), and negative likelihood ratio (-LR) were calculated. DeLong test was used to compare AUC differences between different indicators. Restricted cubic spline (RCS) analysis was conducted to explore the dose-response relationship between cystatin C and AECOPD severity, with four knots set at the 5th, 35th, 65th, and 95th percentiles to test for non-linear relationships (*P* for non-linearity). All statistical tests were two-tailed, with *P* < 0.05 considered statistically significant.

## 3 Results

### 3.1 Baseline characteristics of patients

A total of 389 AECOPD patients were enrolled in this study, comprising 279 males (71.7%) and 110 females (28.3%), with a mean age of 68.71 ± 0.8 years. The average BMI was 22.84 ± 0.1 kg/m^2^. Among the participants, 267 patients (68.6%) had a smoking history, while 142 patients (36.5%) reported alcohol consumption history. Comorbidities included diabetes mellitus in 98 patients (25.2%) and hypertension in 187 patients (48.1%). When patients were stratified by CAT scores, statistically significant differences were observed across groups in terms of age, BMI, systolic blood pressure, smoking history, diabetes mellitus, hypertension, and COPD disease duration (*P* < 0.05). Detailed information is presented in Table 1.

**Table 1:** Comparison of baseline characteristics of patients in different CAT score groups.

Variables	Total (*n* = 389)	Mild (*n* = 82)	Moderate (*n* = 156)	Severe (*n* = 108)	Very severe (*n* = 43)	*F*/χ^2^	*P*
Age, years	68.71 ± 0.8	64.21 ± 0.1	67.81 ± 0.5	71.31 ± 0.2	75.69 ± 0.8	14.28	< 0.001
Male, *n* (%)	279 (71.7)	56 (68.3)	112 (71.8)	78 (72.2)	33 (76.7)	1.12	0.773
BMI, kg/m^2^	22.84 ± 0.1	24.13 ± 0.8	23.24 ± 0.0	22.14 ± 0.2	20.54 ± 0.3	9.87	< 0.001
Systolic blood pressure, mmHg	132.51 ± 8.4	128.61 ± 6.2	131.81 ± 7.9	134.71 ± 9.1	139.22 ± 0.8	4.12	0.007
Diastolic blood pressure, mmHg	78.41 ± 1.2	77.51 ± 0.8	78.21 ± 1.0	78.91 ± 1.6	79.81 ± 2.1	0.54	0.658
Smoke, *n* (%)	267 (68.6)	48 (58.5)	104 (66.7)	80 (74.1)	35 (81.4)	9.45	0.024
Alcohol, *n* (%)	142 (36.5)	28 (34.1)	55 (35.3)	42 (38.9)	17 (39.5)	0.78	0.854
Diabetes, *n* (%)	98 (25.2)	14 (17.1)	36 (23.1)	32 (29.6)	16 (37.2)	8.12	0.044
Hypertension, *n* (%)	187 (48.1)	32 (39.0)	72 (46.2)	56 (51.9)	27 (62.8)	7.89	0.048
Duration of COPD, years	9.25 ± 0.8	6.84 ± 0.2	8.75 ± 0.4	10.86 ± 0.1	13.27 ± 0.1	15.67	< 0.001
Days of hospitalization, days	10.24 ± 0.8	7.53 ± 0.2	9.64 ± 0.1	11.85 ± 0.0	14.66 ± 0.2	23.12	< 0.001

Continuous variables are expressed as x ± s or M (Q_1_, Q_3_), and categorical variables are expressed as n (%).

### 3.2 Laboratory test results

**Figure 1:**
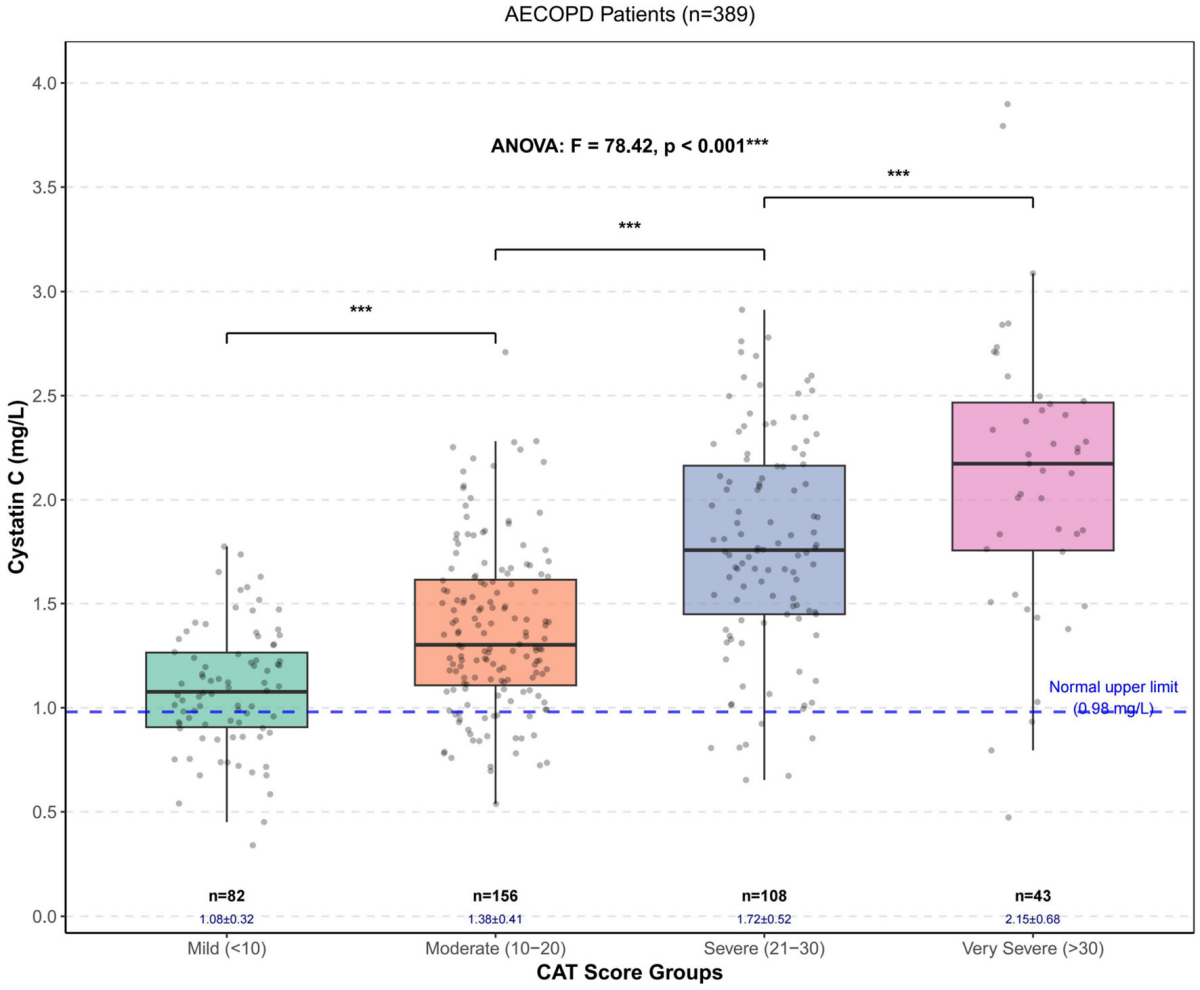
Box plots of cystatin C levels in different CAT score groups.

Comparison of laboratory parameters across different CAT score groups revealed distinct patterns associated with disease severity. Several inflammatory and metabolic markers, including cystatin C, CRP, neutrophil count, D-dimer, fasting glucose, creatinine, blood urea nitrogen, and uric acid levels, showed progressive increases with higher CAT scores. Conversely, absolute lymphocyte count, red blood cell count, hemoglobin, and albumin levels demonstrated significant decreases as CAT scores increased (all *P* < 0.05). Monocyte and platelet counts showed no significant differences between groups (*P*>0.05). Detailed results are presented in Table 2. Cystatin C levels in the overall patient population exhibited a right-skewed distribution, with a median value of 1.42 mg/L and interquartile range of (1.12, 1.78) mg/L, ranging from 0.52 to 3.86 mg/L. Notably, 328 patients (84.3%) had cystatin C levels above the normal upper limit (>0.98 mg/L). Pairwise comparisons revealed statistically significant differences in cystatin C levels between all CAT score groups (*P* < 0.001). Figure 1 illustrates the distribution of cystatin C levels across different CAT score groups. Arterial blood gas parameters showed marked changes corresponding to CAT score severity. As CAT scores increased, pH values and PaO_2_ levels decreased significantly, while PaCO_2_ and lactate levels showed substantial elevations (*P* < 0.001). Additionally, base excess, buffer base, and bicarbonate concentrations demonstrated statistically significant differences between groups (*P* < 0.05). Comprehensive results are detailed in Table 3.

**Table 2:** Comparison of laboratory parameters in patients with different CAT score groups.

Variables	Mild (*n* = 82)	Moderate (*n* = 156)	Severe (*n* = 108)	Very severe (*n* = 43)	*F*/*H*	*P*	Trend *P*-value
NEU, × 10^9^/L	5.822 ± 0.14	7.452 ± 0.68	9.123 ± 0.21	11.564 ± 0.02	42.56	< 0.001	< 0.001
MONO, × 10^9^/L	0.480 ± 0.21	0.520 ± 0.24	0.550 ± 0.26	0.580 ± 0.28	2.18	0.089	0.034
RBC, × 10^9^/L	4.520 ± 0.58	4.380 ± 0.62	4.210 ± 0.68	4.020 ± 0.72	7.89	< 0.001	< 0.001
Hb, g/L	138.51 ± 8.2	132.42 ± 0.1	125.62 ± 2.4	118.22 ± 4.6	11.24	< 0.001	< 0.001
LYM, × 10^9^/L	1.580 ± 0.52	1.360 ± 0.48	1.180 ± 0.44	0.950 ± 0.38	22.67	< 0.001	< 0.001
PLT, × 10^9^/L	218.46 ± 8.5	226.87 ± 2.4	234.27 ± 8.6	242.68 ± 5.2	1.45	0.228	0.067
D-dimer, mg/L	0.68 (0.42, 1.12)	1.24 (0.72, 2.18)	2.15 (1.28, 3.56)	3.42 (2.08, 5.24)	89.45^b^	< 0.001	< 0.001
FPG, mmol/L	5.821 ± 0.45	6.341 ± 0.78	6.892 ± 0.12	7.562 ± 0.48	10.12	< 0.001	< 0.001
Cr, μmol/L	72.41 ± 8.6	82.52 ± 2.4	94.62 ± 8.2	108.43 ± 5.6	23.45	< 0.001	< 0.001
BUN, mmol/L	5.241 ± 0.68	6.422 ± 0.12	7.852 ± 0.56	9.283 ± 0.14	32.18	< 0.001	< 0.001
UA, μmol/L	312.57 ± 8.4	348.69 ± 2.5	385.41 ± 08.2	428.61 ± 25.4	15.67	< 0.001	< 0.001
ALB, g/L	38.64 ± 0.2	36.44 ± 0.8	34.25 ± 0.2	31.55 ± 0.8	24.56	< 0.001	< 0.001
CRP, mg/L	12.4 (6.8, 22.5)	28.6 (15.4, 48.2)	52.4 (32.6, 85.4)	86.5 (58.2, 128.6)	124.56^b^	< 0.001	< 0.001
Cystatin C, mg/L	1.080 ± 0.32	1.380 ± 0.41	1.720 ± 0.52	2.150 ± 0.68	78.42	< 0.001	< 0.001

Continuous variables are expressed as x ± s or M (Q_1_, Q_3_).

NEU, neutrophil; MONO, monocyte; RBC, red blood cell; Hb, hemoglobin; LYM, lymphocyte; PLT, platelet; FPG, fasting plasma glucose; Cr, creatinine; BUN, blood urea nitrogen; UA, uric acid; ALB, albumin; CRP, C-reactive protein.

**Table 3:** Comparison of blood gas analysis results between different CAT score groups.

Variables	Mild (*n* = 82)	Moderate (*n* = 156)	Severe (*n* = 108)	Very severe (*n* = 43)	*F*	*P*	Trend *P*-value
pH	7.35–7.45	7.420 ± 0.04	7.390 ± 0.05	7.360 ± 0.06	7.320 ± 0.08	35.67	< 0.001
PaCO_2_, mmHg	35–45	42.56 ± 0.8	48.68 ± 0.4	56.21 ± 0.5	65.81 ± 4.2	62.34	< 0.001
PaO_2_, mmHg	80–100	72.41 ± 0.2	64.51 ± 1.8	56.21 ± 2.4	48.61 ± 4.6	48.56	< 0.001
BE, mmol/L	−3~+3	1.22 ± 0.8	2.43 ± 0.5	3.84 ± 0.2	5.65 ± 0.1	14.23	< 0.001
BB, mmol/L	45–52	46.83 ± 0.2	48.23 ± 0.8	49.64 ± 0.2	51.44 ± 0.8	15.89	< 0.001
Lac, mmol/L	0.5–1.6	1.240 ± 0.48	1.680 ± 0.62	2.150 ± 0.78	2.861 ± 0.02	56.78	< 0.001
${\text{HCO}}_{3}^{-}$, mmol/L	22–27	25.22 ± 0.8	26.83 ± 0.4	28.43 ± 0.8	30.24 ± 0.5	21.45	< 0.001

BE, base excess; BB, buffered base; Lac, lactic acid; ${\text{HCO}}_{3}^{-}$, bicarbonate ion.

### 3.3 Disease severity assessment results

The CAT scores in the overall patient population ranged from 4 to 38 points, with a mean of 19.28 ± 0.4 points and median of 18 points. Patient distribution across severity groups was as follows: mild group (82 patients, 21.1%), moderate group (156 patients, 40.1%), severe group (108 patients, 27.8%), and very severe group (43 patients, 11.1%). The mMRC dyspnea scale distribution showed: grade 0 (12 patients, 3.1%), grade 1 (77 patients, 19.8%), grade 2 (138 patients, 35.5%), grade 3 (112 patients, 28.8%), and grade 4 (50 patients, 12.9%). There was good concordance between mMRC grading and CAT score groups (Kendall's *W* = 0.72, *P* < 0.001). GOLD staging revealed: stage 1 (28 patients, 7.2%), stage 2 (142 patients, 36.5%), stage 3 (156 patients, 40.1%), and stage 4 (63 patients, 16.2%). The mean FEV_1_ percentage of predicted value was 45.61 ± 6.8%.

Cystatin C levels demonstrated a progressive increase across mMRC dyspnea grades (Table 4). One-way ANOVA revealed highly significant differences between mMRC grades (*F* = 52.36, *P* < 0.001). Cystatin C levels increased stepwise from 0.920 ± 0.24 mg/L in Grade 0 to 2.080 ± 0.65 mg/L in Grade 4, representing a 126% increase. Spearman correlation analysis (Table 5) showed a strong positive correlation between cystatin C and mMRC grading (*rs* = 0.612, 95%CI: 0.545–0.672, *P* < 0.001). Pairwise comparisons with Bonferroni correction revealed statistically significant differences between all grades except between Grade 0 and Grade 1 (*P* = 0.082). Similarly, cystatin C levels showed an increasing trend across GOLD stages (Table 4). One-way ANOVA demonstrated highly significant differences (*F* = 38.45, *P* < 0.001). Cystatin C levels rose from 1.020 ± 0.28 mg/L in GOLD Stage 1 to 1.920 ± 0.62 mg/L in GOLD Stage 4, representing an 88% increase. Spearman correlation analysis (Table 5) revealed a significant positive correlation between cystatin C and GOLD staging (*rs* = 0.534, 95%CI: 0.458–0.602, *P* < 0.001).

**Table 4:** Comparison of cystatin C levels in patients with different mMRC grades and GOLD grades.

Grade	*n* (%)	Cystatin C, mg/L	*F*/*H*	*P*
mMRC grade				
Grade 0	12 (3.1)	0.920 ± 0.24	52.36	< 0.001
Grade 1	77 (19.8)	1.120 ± 0.34		
Grade 2	138 (35.5)	1.380 ± 0.42		
Grade 3	112 (28.8)	1.720 ± 0.54		
Grade 4	50 (12.9)	2.080 ± 0.65		
GOLD grade				
GOLD 1	28 (7.2)	1.020 ± 0.28	38.45	< 0.001
GOLD 2	142 (36.5)	1.280 ± 0.38		
GOLD 3	156 (40.1)	1.560 ± 0.48		
GOLD 4	63 (16.2)	1.920 ± 0.62		

**Table 5:** Spearman correlation analysis between cystatin C and each variable.

Variables	Correlation coefficient (*rs*)	95% CI	*P*
CAT score	0.687	0.628–0.738	< 0.001
mMRC grade	0.612	0.545–0.672	< 0.001
GOLD grade	0.534	0.458–0.602	< 0.001

### 3.4 Correlation analysis

**Figure 2:**
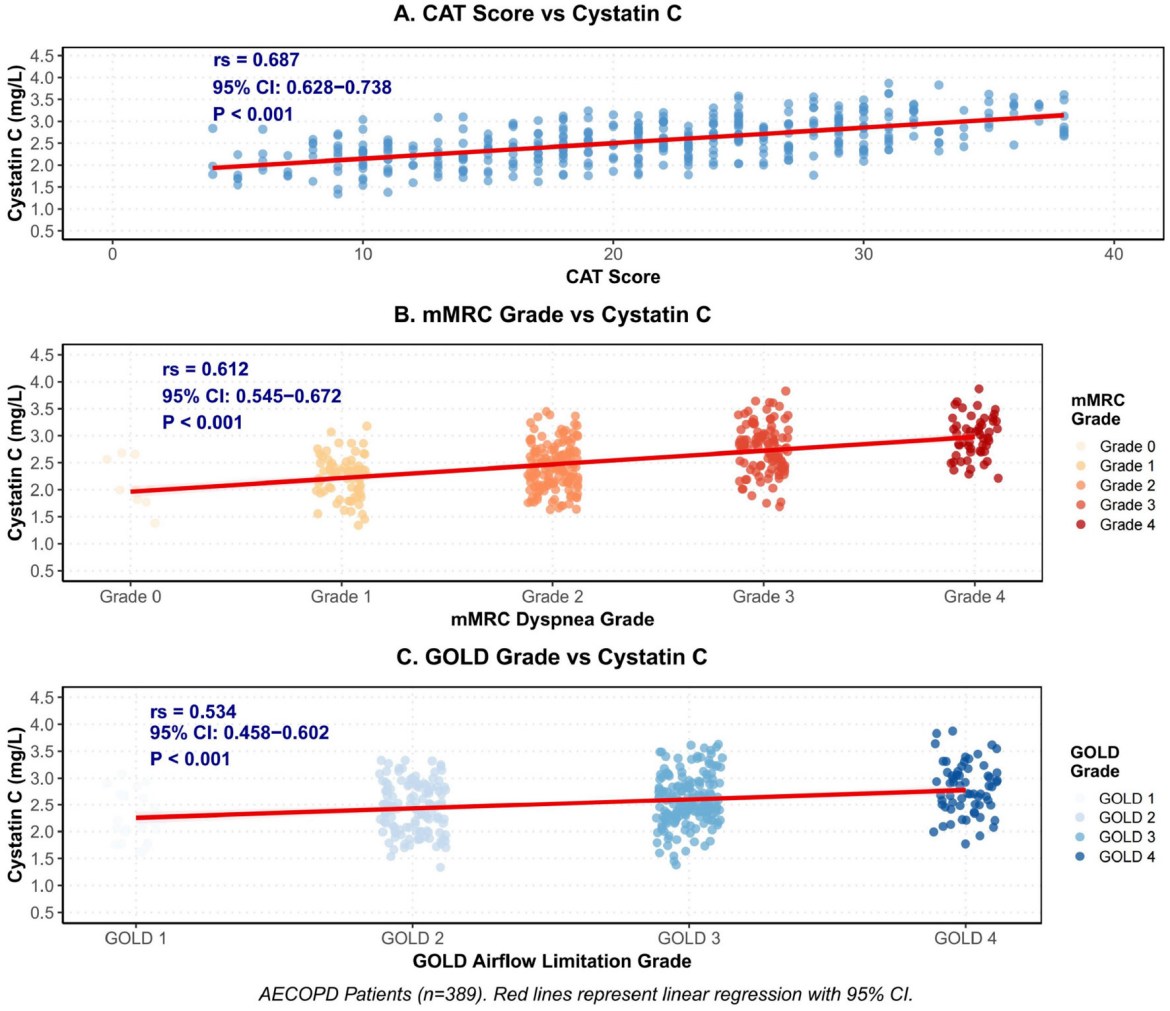
Spearman correlation analysis of cystatin C with CAT score, mMRC grade and GOLD grade. **(A)** CAT Score vs Cystatin C. **(B)** mMRC Grade vs Cystatin C. **(C)** GOLD Grade vs Cystatin C.

Spearman correlation analysis demonstrated strong positive associations between cystatin C and multiple disease severity indicators. Cystatin C showed a strong positive correlation with CAT scores (*rs* = 0.687, *P* < 0.001) and mMRC dyspnea grading (*rs* = 0.612, *P* < 0.001), while exhibiting a moderate positive correlation with GOLD staging (*rs* = 0.534, *P* < 0.001). These findings are detailed in Table 5 and illustrated in Figure 2.

**Figure 3:**
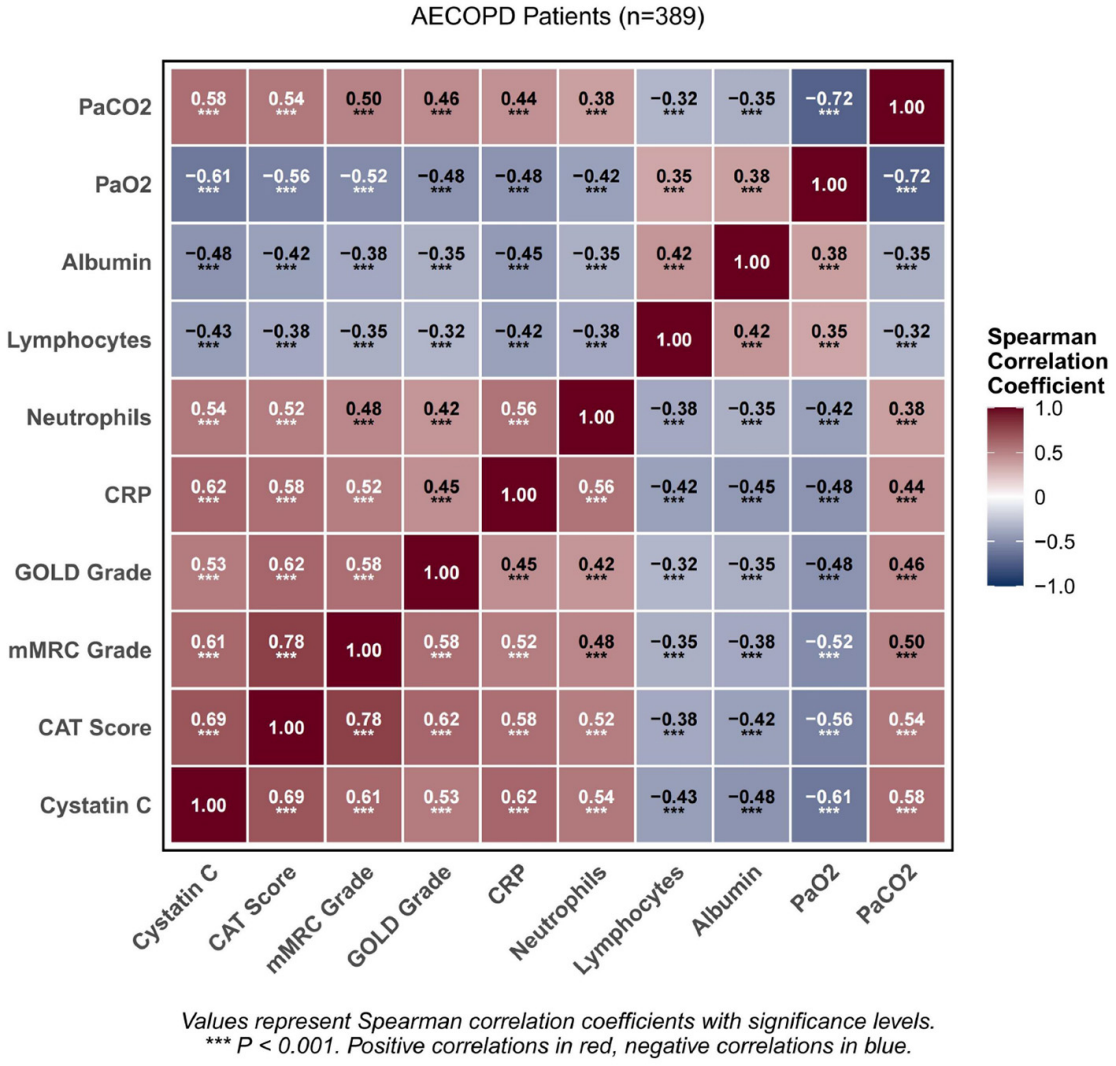
Presents the heatmap of correlations between the primary variables.

To evaluate the independence of these associations from potential confounding factors, partial correlation analysis was performed after controlling for age, gender, BMI, smoking history, diabetes, hypertension, and creatinine levels. The results revealed that cystatin C maintained significant correlations with disease severity measures: CAT scores (partial correlation coefficient = 0.542, *P* < 0.001), mMRC grading (partial correlation coefficient = 0.486, *P* < 0.001), and GOLD staging (partial correlation coefficient = 0.412, *P* < 0.001). These findings indicate that the association between cystatin C and disease severity is independent of the aforementioned confounding variables. Figure 3 presents a correlation heatmap of key variables, which provides a comprehensive visualization of the relationships among study parameters. The heatmap clearly demonstrates that cystatin C exhibits positive correlations with disease severity indicators (CAT, mMRC, GOLD) and inflammatory markers (CRP, neutrophil count), while showing negative correlations with nutritional status indicators (albumin) and oxygenation parameters (PaO_2_).

### 3.5 Multiple linear regression analysis

Multiple stepwise regression analysis was performed with CAT score as the dependent variable, incorporating all variables with *P* < 0.05 from univariate analysis, including age, BMI, smoking history, diabetes, hypertension, COPD duration, cystatin C, CRP, neutrophil count, lymphocyte count, albumin, creatinine, blood urea nitrogen, D-dimer, PaO_2_, PaCO_2_, and lactate. The final model retained six variables and demonstrated good predictive performance with an adjusted *R*^2^ = 0.628 (*F* = 108.56, *P* < 0.001). Notably, cystatin C emerged as the strongest independent predictor of CAT scores (β = 5.89, *P* < 0.001). Detailed results are presented in Table 6.

**Table 6:** Multiple linear regression analysis of influencing factors of CAT score.

Variables	Non-normalized factor *B*	Standard error, SE	Standardized coefficient β	T	*P*	95%CI	VIF
Constant term	8.24	2.56	—	3.22	0.001	3.21 to 13.27	—
Cystatin C	5.89	0.59	0.382	9.98	< 0.001	4.72 to 7.06	1.86
CRP (lg transformed)	3.42	0.48	0.268	7.13	< 0.001	2.48 to 4.36	1.72
PaO_2_	−0.12	0.02	−0.198	−5.24	< 0.001	−0.17 to −0.08	1.45
Age	0.09	0.03	0.118	3.12	0.002	0.03 to 0.15	1.32
ALB	−0.28	0.08	−0.142	−3.68	< 0.001	−0.43 to −0.13	1.38
Duration of COPD	0.18	0.06	0.108	2.86	0.004	0.06 to 0.31	1.28

Model statistics: adjusted R^2^ = 0.628, F = 108.56, P < 0.001, Durbin–Watson = 1.92.

### 3.6 Logistic regression analysis

To evaluate the independent predictive value of cystatin C for severe AECOPD, binary logistic regression analysis was performed with severe AECOPD as the dependent variable. Three progressively adjusted models were constructed to account for potential confounding factors. Cystatin C consistently emerged as an independent risk factor for severe AECOPD across all models (Table 7). In the fully adjusted model (Model 3), each 1 mg/L increase in cystatin C was associated with a 4.28-fold increased risk of severe AECOPD (OR = 5.28, 95% CI: 3.12–8.94, *P* < 0.001). When cystatin C was stratified into quartile categories (< 1.2 mg/L, 1.2–1.6 mg/L, 1.6–2.0 mg/L, and ≥2.0 mg/L) with < 1.2 mg/L as the reference group, Model 3 demonstrated a dose-dependent relationship between cystatin C levels and severe AECOPD risk. The risk of severe AECOPD increased significantly with each higher cystatin C stratum, reaching a 7.92-fold increased risk in patients with cystatin C levels ≥2.0 mg/L compared to the reference group.

**Table 7:** Binary logistic regression analysis of risk factors for severe AECOPD.

Variables	Model 1 (single factor)		Model 2 (adjusted demographics)		Model 3 (fully adjusted)	
	**OR (95%CI)**	***P***	**OR (95%CI)**	***P***	**OR (95%CI)**	***P***
Cystatin C (per 1 mg/L increase)	6.84 (4.25–11.02)	< 0.001	6.12 (3.76–9.96)	< 0.001	5.28 (3.12–8.94)	< 0.001
Cystatin C stratification						
< 1.2 mg/L	1.00 (Reference)	—	1.00 (Reference)	—	1.00 (Reference)	—
1.2–1.6 mg/L	2.86 (1.68–4.87)	< 0.001	2.64 (1.52–4.58)	0.001	2.42 (1.36–4.31)	0.003
1.6–2.0 mg/L	5.42 (3.08–9.54)	< 0.001	4.89 (2.72–8.78)	< 0.001	4.26 (2.28–7.96)	< 0.001
≥2.0 mg/L	12.56 (6.42–24.58)	< 0.001	10.86 (5.42–21.76)	< 0.001	8.92 (4.28–18.60)	< 0.001
Trend *P-*value	< 0.001		< 0.001		< 0.001	

Model 1: univariate analysis.

Model 2: adjusted for age, sex, BMI.

Model 3: further adjusted for smoking history, diabetes, hypertension, CRP, creatinine, PaO, ALB. Hosmer-Lemeshow test: χ^2^ = 6.84, P = 0.554, good model fit.

### 3.7 ROC curve analysis

**Figure 4:**
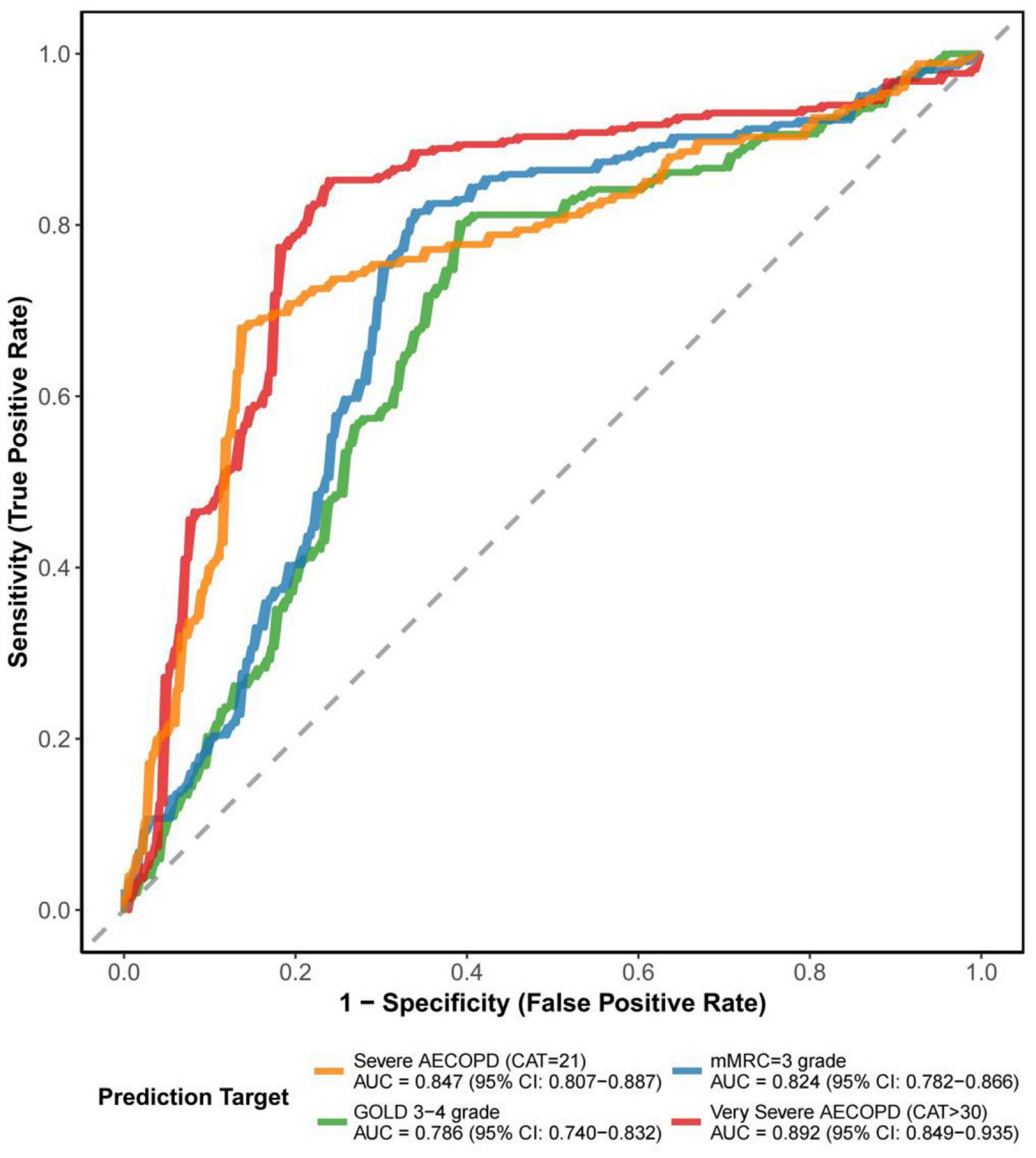
ROC curve for cystatin C in predicting AECOPD severity.

ROC curves were constructed to evaluate the predictive performance of cystatin C for different severities of AECOPD. The results demonstrated good discriminatory ability across multiple severity classifications. Cystatin C achieved an AUC of 0.847 (95% CI: 0.807–0.887) for predicting severe AECOPD (CAT ≥ 21 points), 0.824 (95% CI: 0.782–0.866) for predicting mMRC ≥ Grade 3, 0.786 (95% CI: 0.740–0.832) for predicting GOLD Stages 3–4, and 0.892 (95% CI: 0.849–0.935) for predicting very severe AECOPD (CAT > 30 points). Comprehensive results are presented in Table 8 and Figure 4.

**Table 8:** ROC curve analysis of cystatin C in predicting the severity of AECOPD.

Predicted target	AUC	95% CI	Cut-off	Sensitivity	Specificity	PPV	NPV	+LR	-LR	Youden index
Severe AECOPD (CAT ≥ 21)	0.847	0.807–0.887	1.52	81.5%	78.2%	70.4%	86.8%	3.74	0.24	0.597
mMRC ≥ 3 grade	0.824	0.782–0.866	1.48	78.4%	76.8%	68.2%	84.6%	3.38	0.28	0.552
GOLD 3–4 grade	0.786	0.740–0.832	1.42	74.6%	72.4%	75.8%	71.0%	2.70	0.35	0.470
Very severeAECOPD (CAT > 30)	0.892	0.849–0.935	1.86	83.7%	82.4%	52.8%	95.6%	4.76	0.20	0.661

PPV, positive predictive value; NPV, negative predictive value; +LR, positive likelihood ratio; -LR, negative likelihood ratio.

**Figure 5:**
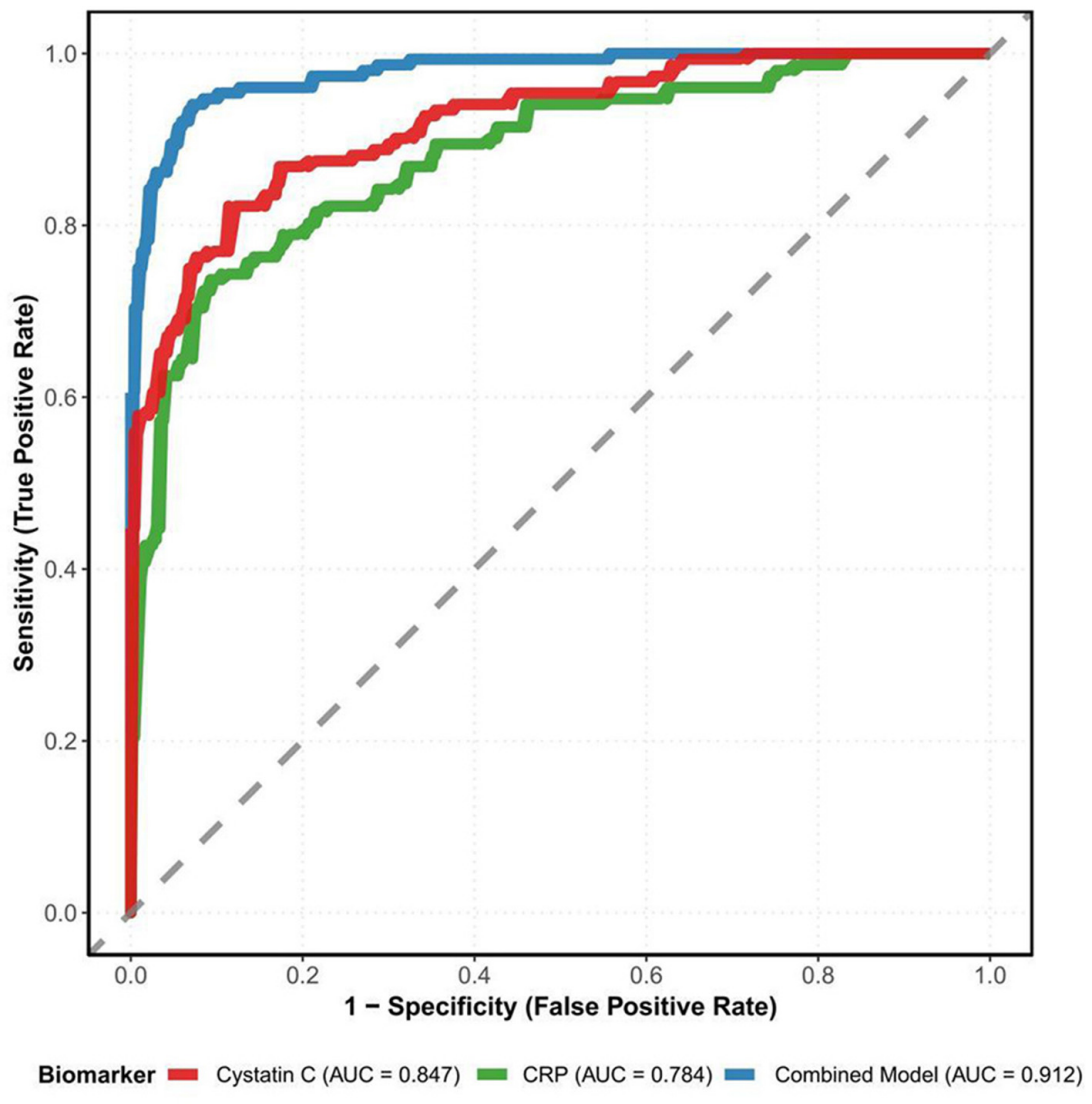
Comparison of ROC curves of different indicators for predicting severe AECOPD.

DeLong test was employed to compare the diagnostic performance of cystatin C with CRP, creatinine, and combined biomarkers for predicting severe AECOPD. Cystatin C demonstrated significantly superior diagnostic performance compared to both CRP (AUC: 0.847 vs. 0.784, *Z* = 2.86, *P* = 0.004) and creatinine (AUC: 0.847 vs. 0.698, *Z* = 5.42, *P* < 0.001). Notably, the combination of cystatin C with CRP and PaO_2_ achieved an AUC of 0.912 (95% CI: 0.881–0.943), which was significantly higher than cystatin C alone (*Z* = 3.68, *P* < 0.001). Detailed comparative analyses are shown in Table 9 and Figure 5.

**Table 9:** Comparison of ROC curves of different indicators for predicting severe AECOPD.

Variables	AUC	95% CI	Comparison with cystatin C	
			***Z***	***P***
Cystatin C	0.847	0.807–0.887	—	—
CRP	0.784	0.738–0.830	2.86	0.004
Creatinine	0.698	0.646–0.750	5.42	< 0.001
BUN	0.712	0.661–0.763	4.86	< 0.001
NEU	0.756	0.708–0.804	3.52	< 0.001
PaO_2_	0.802	0.758–0.846	1.82	0.069
Combined measures^a^	0.912	0.881–0.943	3.68^b^	< 0.001

Combined measures^*a*^: cystatin C + CRP + PaO.

### 3.8 Restrictive cubic spline analysis

**Figure 6:**
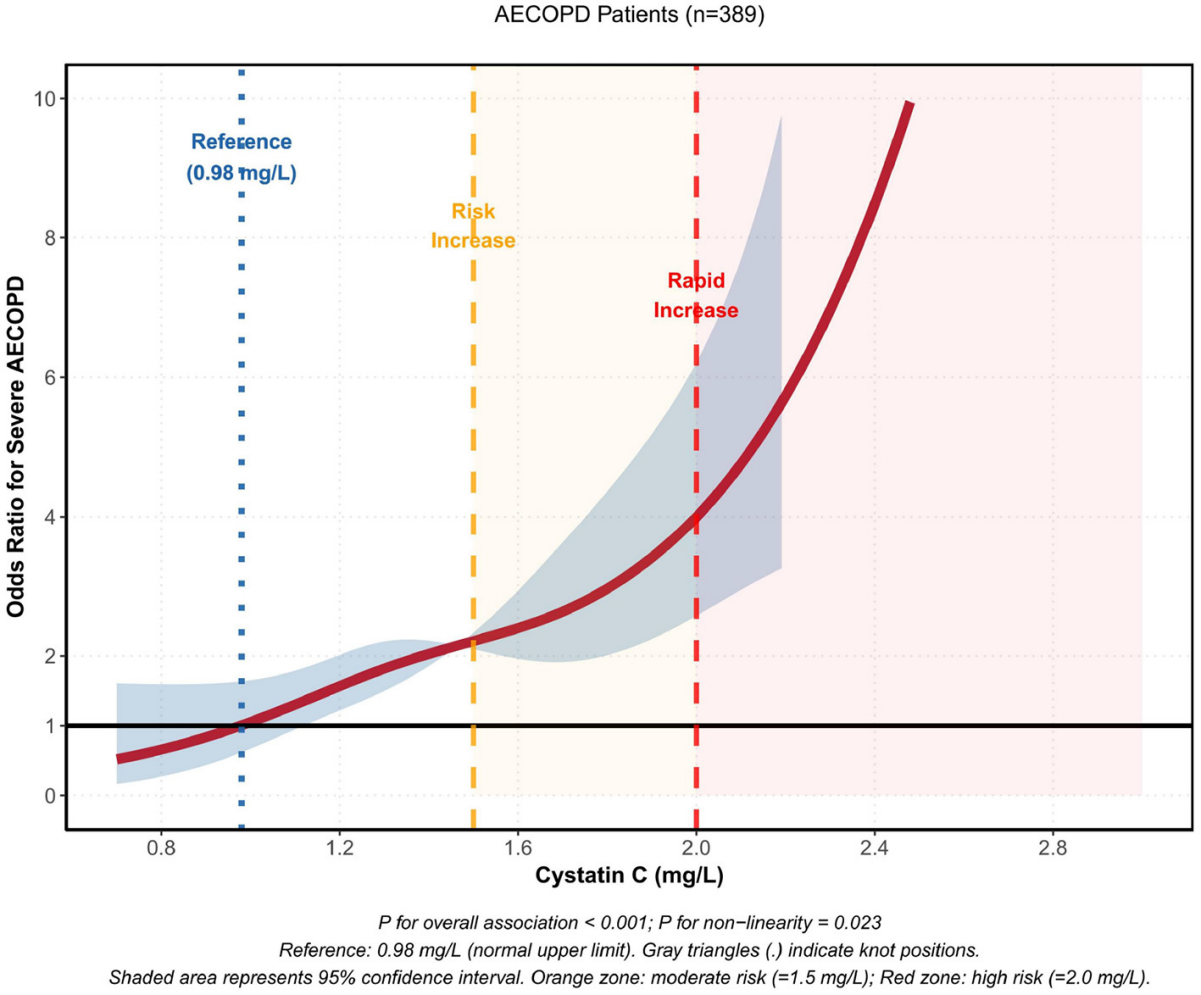
Restrictive cubic spline (RCS) analysis to explore the dose-response relationship between cystatin C and risk of severe AECOPD.

Restricted cubic spline (RCS) analysis was performed to explore the dose-response relationship between cystatin C levels and the risk of severe AECOPD. Four knots were positioned at the 5th, 35th, 65th, and 95th percentiles, corresponding to cystatin C levels of 0.82, 1.28, 1.68, and 2.42 mg/L, respectively. The analysis revealed a significant non-linear relationship between cystatin C and severe AECOPD risk (*P* for non-linearity = 0.023), with the overall association being highly significant (*P* for overall < 0.001). Using the normal upper limit of cystatin C (0.98 mg/L) as the reference point, the risk of severe AECOPD increased significantly when cystatin C levels reached ≥1.5 mg/L, and demonstrated a sharp escalation when levels exceeded ≥2.0 mg/L. The dose-response curve is illustrated in Figure 6.

## 4 Discussion

Acute exacerbations of chronic obstructive pulmonary disease (AECOPD) represent critical events in the disease trajectory of COPD patients, closely associated with accelerated lung function decline, deteriorating quality of life, and significantly increased mortality risk (15). The identification of biomarkers capable of early recognition of high-risk patients and effective assessment of disease severity holds paramount importance for optimizing clinical decision-making and improving outcomes. This study systematically evaluated the relationships between serum cystatin C (CysC) and symptom burden, airflow limitation severity, and clinical severity among 389 hospitalized AECOPD patients. Our findings demonstrate that CysC exhibits an independent, robust, and non-linear positive correlation with AECOPD severity, suggesting its potential as a clinically translatable risk stratification biomarker in this population.

The AECOPD patients included in this study exhibited typical demographic and clinical characteristics: pre-dominantly elderly males (mean age 68.7 years, 71.7% male), high smoking exposure rates (68.6%), and substantial comorbidity burden (25.2% diabetes, 48.1% hypertension). These characteristics are consistent with reports from multiple large-scale COPD cohort studies both domestically and internationally (16, 17). Notably, when stratified by CAT scores, significant differences emerged across groups in terms of age, BMI, smoking history, diabetes, hypertension, and COPD duration, indicating that higher symptom burden does not exist in isolation but accompanies advanced age, poorer nutritional status, longer disease duration, and increased cardiovascular-metabolic comorbidities. This “high symptom burden—high comorbidity load” phenotypic clustering phenomenon aligns with the GOLD guidelines' emphasis on COPD heterogeneity and multidimensional assessment (15), while providing necessary justification for controlling confounding factors in subsequent analyses.

Our study revealed that as CAT score stratification increased, levels of CysC, CRP, neutrophil count, D-dimer, fasting glucose, creatinine, blood urea nitrogen, and uric acid showed synchronous increasing trends, while absolute lymphocyte count, red blood cell count, hemoglobin, and albumin levels demonstrated declining patterns. These findings suggest that AECOPD patients with heavier symptom burden may exist in states of more intense systemic inflammatory response, more pronounced pro-coagulant/endothelial activation, and poorer nutritional and anemic conditions. This observation aligns with previous research: Agusti et al. (18) proposed the “systemic inflammatory syndrome” hypothesis of COPD, arguing that persistent systemic inflammation serves as the core mechanism driving extrapulmonary comorbidities and adverse outcomes. Vanfleteren et al. (19) further confirmed that inflammatory-metabolic-cardiovascular risk factors commonly exhibit clustered distribution in COPD patients, collectively shaping phenotypic heterogeneity and prognostic differences.

CysC, as a low-molecular-weight protein constitutively secreted by all nucleated cells, is traditionally regarded as a sensitive marker of glomerular filtration function, with serum levels less influenced by muscle mass, age, and gender compared to creatinine (20). However, accumulating evidence suggests that the clinical significance of CysC extends beyond simple renal function assessment. Shlipak et al. (21) confirmed in a prospective cohort study of over 11,000 adults that elevated CysC was independently associated with all-cause mortality, cardiovascular events, and heart failure risk, with predictive efficacy superior to creatinine-based renal function estimation. In our study, the median CysC level among all AECOPD patients was 1.42 mg/L, with 84.3% having levels above the normal upper limit, and highly significant statistical differences observed in pairwise comparisons of CysC levels between all CAT groups, suggesting that CysC elevation has extremely high occurrence rates and potential stratification value in this population. The correlation heatmap further revealed that CysC positively correlated with inflammatory markers (CRP, neutrophils) while negatively correlating with nutritional markers (albumin) and oxygenation parameters (PaO_2_), indicating that CysC may be positioned at the convergence node of the “inflammation-hypoxia-nutritional depletion-renal vulnerability” pathological network, rather than reflecting a single pathophysiological pathway.

Arterial blood gas analysis results showed that as CAT scores increased, patient pH values and PaO_2_ decreased significantly, while PaCO_2_ and lactate levels increased substantially, accompanied by changes in acid-base compensation-related indicators. This finding confirms the tight coupling between higher symptom burden and more severe ventilation/gas exchange dysfunction and tissue perfusion/metabolic stress. Hypoxemia and hypercapnia are not only core pathophysiological features of AECOPD but also important predictors of poor prognosis (15, 22). Samareh Fekri et al. (23) reported that the PaO_2_/FiO_2_ ratio in AECOPD patients was significantly negatively correlated with in-hospital mortality, while hypercapnia was associated with increased risk of mechanical ventilation requirements and ICU admission.

The negative correlation between CysC and PaO_2_ observed in our study deserves in-depth discussion. On one hand, persistent or recurrent hypoxia can impair renal function through pathways including activation of the renin-angiotensin-aldosterone system, induction of renal tubular ischemia, and oxidative stress injury, leading to CysC elevation. On the other hand, renal function decline itself can exacerbate respiratory function and oxygenation status through mechanisms such as increased volume load, worsened anemia, and impaired inflammatory mediator clearance, forming a vicious cycle of “heart-lung-kidney” interactions (24). Although the cross-sectional design of this study cannot establish causal direction, the above correlation structure provides biological plausibility for CysC as a comprehensive pathophysiological status marker.

This study employed three complementary severity assessment tools: CAT score, mMRC grading, and GOLD staging. CAT score comprehensively reflects patients' symptom burden and health status impairment, mMRC grading focuses on dyspnea as a core symptom, while GOLD staging objectively quantifies airflow limitation based on FEV1 percentage of predicted value (15). The results showed good concordance between mMRC grading and CAT score groups (Kendall's W = 0.72), confirming the synergy of these two subjective assessment tools in reflecting disease impact.

More importantly, CysC showed significant positive correlations with all three severity indicators: the highest correlation coefficient with CAT scores (*rs* = 0.687), followed by mMRC grading (*rs* = 0.612) and GOLD staging (*rs* = 0.534). After controlling for potential confounding factors including age, gender, BMI, smoking history, diabetes, hypertension, and creatinine, partial correlation coefficients decreased but remained highly statistically significant (partial correlation coefficients of 0.542, 0.486, and 0.412, respectively). This result holds important significance: first, it indicates that the association between CysC and disease severity is not entirely explained by renal function (approximated by creatinine) or other common confounding factors, demonstrating certain independence; second, it suggests that CysC may integrate multidimensional pathophysiological information rather than merely reflecting a single dimension of disease status.

Zhang et al. (25) also reported a positive correlation between CysC and GOLD staging in a study of 426 stable COPD patients, but their correlation coefficient (*rs* = 0.38) was lower than our findings. This difference may be related to different study populations (stable vs. acute exacerbation) and disease states. During acute exacerbations, inflammatory flares, enhanced oxidative stress, and multi-organ functional fluctuations may amplify the association strength between CysC and disease severity a hypothesis that awaits validation by prospective studies.

Multiple stepwise regression analysis incorporated 17 variables with statistical significance from univariate analysis into the model, ultimately selecting 6 variables for the final model with an adjusted *R*^2^ = 0.628, indicating good model explanation for CAT score variation. Notably, CysC emerged as the strongest independent predictor of CAT scores (β = 5.89), with its standardized regression coefficient ranking first among all selected variables. This finding suggests that after controlling for other clinical and laboratory indicators, CysC's explanatory contribution to symptom burden holds a prominent position.

Previous studies have primarily focused on the relationship between traditional inflammatory markers such as CRP and fibrinogen with COPD severity (26, 27). Thomsen et al. (28) confirmed in the Copenhagen City Heart Study that persistently elevated CRP, fibrinogen, and white blood cell count were independently associated with increased COPD acute exacerbation frequency. Our study, while incorporating CRP and other indicators, found that CysC possessed superior predictive efficacy, suggesting that CysC may capture pathophysiological information not fully reflected by traditional inflammatory markers, such as subclinical renal function impairment, microvascular dysfunction, or tissue hypoxia severity.

Logistic regression analysis constructed three progressively adjusted models to evaluate the independent predictive value of CysC for severe AECOPD (CAT ≥ 21 points). Results showed that CysC was an independent risk factor for severe AECOPD across all models. In the fully adjusted model controlling for demographic characteristics, comorbidity status, inflammatory markers, and renal function (Model 3), each 1 mg/L increase in CysC was associated with a 4.28-fold increased risk of severe AECOPD (OR = 5.28, 95% CI: 3.12–8.94). Stratified analysis further revealed a clear dose-response relationship: using CysC < 1.2 mg/L as reference, the risk in groups with 1.2–1.6 mg/L, 1.6–2.0 mg/L, and ≥2.0 mg/L increased sequentially, with the ≥2.0 mg/L group showing a 7.92-fold increased risk. This stable “independence + gradient” association pattern holds important translational significance in clinical practice. It suggests that CysC can be used to identify AECOPD patients on high-risk trajectories early during admission, thereby assisting clinicians in formulating more aggressive monitoring and intervention strategies. For example, patients with CysC ≥ 2.0 mg/L might be considered for earlier initiation of non-invasive ventilatory support, more intensive blood gas monitoring, or more timely ICU transfer evaluation.

ROC curve analysis systematically evaluated the discriminatory efficacy of CysC for different severities of AECOPD. Results showed that CysC achieved an AUC of 0.847 for predicting severe AECOPD (CAT ≥ 21 points), 0.824 for predicting mMRC ≥ Grade 3, 0.786 for predicting GOLD Stages 3–4, and 0.892 for predicting very severe AECOPD (CAT > 30 points). These results indicate that CysC possesses good discriminatory ability for severe disease states under multiple definitions, with particularly outstanding identification capability for very severe cases. DeLong testing further compared the diagnostic efficacy of CysC with traditional markers. Results showed that CysC's AUC was significantly higher than CRP (0.847 vs. 0.784, *P* = 0.004) and creatinine (0.847 vs. 0.698, *P* < 0.001), demonstrating superior single-indicator predictive capability compared to these commonly used indicators. This finding echoes research conclusions by Shlipak et al. (21) in cardiovascular disease populations, namely that CysC possesses superior risk prediction value compared to creatinine.

More importantly, the combination of CysC with CRP and PaO_2_ increased the AUC to 0.912, significantly higher than CysC alone (*P* < 0.001). This result suggests that integrating multidimensional information reflecting inflammatory intensity (CRP), oxygenation impairment (PaO_2_), and organ function/systemic stress (CysC) can significantly enhance severity identification capability. This aligns highly with the pathophysiological framework where “inflammatory flare + gas exchange impairment + multi-organ interactive injury” collectively determine clinical severity during acute exacerbations (2, 15). Future research can further explore integrating these indicators into composite scores or nomograms to optimize their clinical practicality.

Restricted cubic spline (RCS) analysis is a powerful tool for exploring dose-response relationships between continuous variables and outcomes, avoiding information loss or bias introduction from artificially set classification thresholds (29). Our study employed RCS analysis and found a significant non-linear relationship between CysC and severe AECOPD risk (*P* for non-linearity = 0.023), with the overall association being highly statistically significant (*P* for overall < 0.001). However, it is crucial to interpret the clinical implications of this identified threshold with caution. As these cut-offs are data-driven and derived from a specific population, their direct applicability in diverse clinical settings requires rigorous external validation in larger and independent cohorts before widespread clinical implementation. While this threshold offers a promising initial guide for risk stratification, further research is warranted to confirm its generalizability and clinical utility across different ethnicities, regions, and healthcare settings.

Using CysC = 0.98 mg/L (normal upper limit) as reference, the risk curve exhibited obvious threshold characteristics: when CysC < 1.5 mg/L, risk increase was relatively gradual; when CysC ≥ 1.5 mg/L, risk began to rise significantly; when CysC ≥ 2.0 mg/L, the risk curve accelerated sharply. This non-linear pattern holds important clinical implications: first, it suggests the existence of a “risk acceleration zone,” where CysC elevation above a certain threshold brings far greater risk increments than below the threshold; second, it provides statistical basis for setting warning thresholds in clinical practice, for example, establishing CysC ≥ 1.5 mg/L as a “warning value” and ≥2.0 mg/L as a “high-risk value” for stratified management. It's worth noting that the thresholds determined in this study are based on a single-center sample, and their external validity requires validation in populations of different regions, ethnicities, and healthcare settings. Additionally, optimal threshold selection should comprehensively consider sensitivity-specificity trade-offs in clinical practice as well as intervention accessibility and cost-effectiveness.

This study successfully established a strong correlation between serum cystatin C and CAT scores in AECOPD patients, as well as its diagnostic efficacy for severe AECOPD. However, we also need to interpret the clinical implications based on CAT scores with caution. We acknowledge that the CAT score, as a patient-reported outcome measure, has a certain degree of subjectivity and can be influenced by acute symptoms, cultural factors, and individual perception differences. Therefore, its clinical application in predicting cystatin C-defined severity should be interpreted carefully. Nevertheless, the CAT score is a widely accepted and highly practical tool in the field of COPD, comprehensively reflecting the patient's symptom burden and health status. It is crucial for assessing the real impact of the disease on patients' daily lives. The significant association between cystatin C and CAT scores in this study precisely highlights the potential of this objective biomarker in capturing the subjectively perceived severity of the disease by patients, thereby providing clinicians with a quantifiable and readily available tool to supplement existing assessments.

The biological mechanisms underlying the association between CysC and AECOPD severity may involve multiple levels. First, as a member of the cysteine protease inhibitor superfamily, CysC participates in regulating cathepsin activity, which plays important roles in lung tissue remodeling, elastin degradation, and airway inflammatory responses (30). Elevated circulating CysC may reflect compensatory increases from local consumption or activation of systemic anti-protease responses. Second, as previously discussed, CysC elevation may reflect subclinical or overt renal function impairment during acute exacerbations. COPD patients commonly possess multiple risk factors for renal function impairment, including advanced age, smoking, diabetes, hypertension, and repeated use of nephrotoxic medications (31). During acute exacerbations, hypoxemia, hemodynamic instability, and systemic inflammation may further exacerbate renal hypoperfusion and injury. Chen et al. (32) found in a retrospective study of 2,500 AECOPD patients that reduced estimated glomerular filtration rate (eGFR) at admission was independently associated with increased in-hospital mortality, supporting the “heart-lung-kidney interactive injury” hypothesis. Third, CysC itself may participate in inflammatory regulation. *In vitro* studies indicate that CysC can modulate inflammatory cytokine secretion by monocytes/macrophages and influence neutrophil chemotaxis and apoptosis (33). Under systemic inflammatory conditions, CysC expression and secretion may be altered, and its circulating levels may therefore partially reflect the degree of inflammatory activation. The positive correlations between CysC and CRP, neutrophil count observed in our study support this hypothesis.

Building upon the validated significant positive correlation between cystatin C and AECOPD severity, this study further contributes several important new clinical insights. Firstly, through a cohort study of 389 hospitalized AECOPD patients, we strengthened and expanded the evidence base for cystatin C as a biomarker in this specific high-risk population. Compared to previous studies with smaller sample sizes or those not focusing on acute exacerbations, our findings possess greater clinical representativeness and generalizability. Secondly, this study first clarified that serum cystatin C exhibits significant diagnostic efficacy in predicting severe AECOPD (defined as a CAT score > 20 points), establishing an optimal diagnostic cut-off value of 1.52 mg/L, with a sensitivity of 81.5% and specificity of 78.2% (AUC = 0.847). This crucial finding elevates cystatin C from a mere “associational biomarker” to a “tool” with practical clinical diagnostic value. This implies that in clinical practice, when the serum cystatin C level of a hospitalized AECOPD patient exceeds this threshold, a severe disease state should be highly suspected, and more aggressive interventions considered. This specific and actionable cut-off value addresses the current limitations in the objectivity of existing clinical assessment methods, providing clinicians with a basis for earlier and more accurate identification of high-risk AECOPD patients. Furthermore, cystatin C's role as an independent predictor of CAT scores further highlights its unique value in assessing AECOPD severity within a multi-factorial context. This independence suggests that cystatin C not only reflects known pathophysiological processes but may also capture information about disease severity that other traditional indicators fail to adequately reflect. Collectively, these findings suggest that incorporating serum cystatin C into the routine assessment process for AECOPD has the potential to optimize current clinical assessment practices, aid in clinical decision-making, facilitate early identification and individualized management of high-risk patients, and ultimately improve patient outcomes.

This study has several limitations that warrant objective acknowledgment. Firstly, the inherent cross-sectional design, coupled with cystatin C measurements taken solely at hospital admission, limits our ability to infer causality or predict the progression of AECOPD severity. Our findings primarily demonstrate an association between cystatin C levels and disease severity, rather than establishing cystatin C as a predictive or causative factor. It is not possible to determine from this study whether elevated cystatin C contributes to exacerbation severity or merely reflects the ongoing complex pathophysiological changes (e.g., inflammation, hypoxia, or subclinical renal dysfunction) during the acute phase. Furthermore, single CysC measurements cannot reflect dynamic change trajectories, nor distinguish between acute-phase fluctuations and chronic baseline levels. Future longitudinal studies with serial measurements of cystatin C throughout the exacerbation course are needed to delineate its dynamic role, predictive capacity, and potential causal links with AECOPD severity and outcomes. Secondly, the single-center and retrospective design, coupled with the exclusive inclusion of hospitalized AECOPD patients, introduces potential selection bias and significantly limits the generalizability of our findings. The study population likely represents a more severe spectrum of AECOPD, and thus our results, including the identified diagnostic thresholds, may not be applicable to patients with milder exacerbations or those managed in outpatient settings. Future multicenter prospective studies encompassing a broader range of AECOPD severity and care settings are necessary to validate these findings and enhance their external validity. Thirdly, a significant limitation inherent in using cystatin C as a biomarker is its close association with renal function. While we adjusted for serum creatinine in our analysis, the possibility that the observed associations between cystatin C levels and AECOPD severity primarily reflect acute or subclinical renal dysfunction during AECOPD cannot be entirely excluded. Acute exacerbations often involve systemic inflammation, hypoxemia, and hemodynamic instability, all of which can affect renal perfusion and function. Therefore, the elevated cystatin C levels might be a composite indicator reflecting both the severity of the exacerbation and a co-occurring subtle impairment of renal function. Future studies incorporating more comprehensive assessments of renal function (e.g., direct GFR measurements or novel renal biomarkers) are warranted to better delineate the independent contributions of AECOPD severity and renal dysfunction to cystatin C levels. Fourthly, single CysC measurements cannot reflect dynamic change trajectories, nor distinguish between acute-phase fluctuations and chronic baseline levels; future studies could employ continuous measurements at admission and before discharge to evaluate the relationship between CysC change magnitude and prognosis. Fifthly, the main endpoint of this study was disease severity defined by CAT scores, representing a surrogate endpoint; CysC's predictive value for hard endpoints (such as in-hospital mortality, ICU admission, mechanical ventilation requirements, 30-day readmission, etc.) awaits further validation. Finally, the CysC thresholds determined in this study are based on a specific population, and their applicability across different ethnicities, regions, and healthcare settings requires confirmation through external validation studies.

This study confirms that serum cystatin C is closely related to disease severity indicators (including CAT score, mMRC grading, and GOLD staging) in hospitalized AECOPD patients and is an independent predictor of CAT scores. More importantly, this study is the first to establish and validate the optimal diagnostic cut-off value (1.52 mg/L) of serum cystatin C for predicting severe AECOPD in a large cohort of hospitalized AECOPD patients, demonstrating good diagnostic sensitivity and specificity. These results suggest that serum cystatin C is not only a strongly correlated biomarker but also a diagnostic tool with direct clinical application value, which is expected to significantly enhance clinical assessment and management strategies for AECOPD by providing objective and quantitative risk assessment.

## Data Availability

The original contributions presented in the study are included in the article/supplementary material, further inquiries can be directed to the corresponding author.
