# Correction: Feasibility of cystatin C as a biomarker for AECOPD severity: a cross-sectional study integrating CAT score, mMRC grade, and GOLD stage

**DOI:** 10.3389/fmed.2026.1884988

**Published:** 2026-06-03

**Authors:** Haoran Ma, Mengwen Yan, Jun Liu, Chen Zhu

**Affiliations:** 1 Department of Respiratory and Critical Care Medicine, Fuyang Women's and Children's Hospital, Fuyang, Anhui, China; 2 Department of Ophthalmology, Fuyang Women's and Children's Hospital, Fuyang, Anhui, China; 3 Department of Obstetrics and Gynecology, Fuyang Women's and Children's Hospital, Fuyang, Anhui, China

**Keywords:** acute exacerbations of chronic obstructive pulmonary disease, biomarker, CAT score, cystatin C, GOLD staging, mMRC grading

There was a mistake in Figures 4, 5 as published.

Figure 4 (ROC Curve Analysis), the legend describing the color coding of the four curves was swapped during the proof stage. The current published legend misrepresents which color corresponds to which clinical endpoint.

The correct mapping is:

Orange line: Severe AECOPD (CAT = 21), AUC = 0.847.

Blue line: mMRC = 3 grade, AUC = 0.824.

Green line: GOLD 3–4 grade, AUC = 0.786.

Red line: Very Severe AECOPD (CAT > 30), AUC = 0.892.

Figure 5 (ROC curve analysis), the legend describing the color coding of the three curves was swapped during the proof stage. The current published legend misrepresents which color corresponds to which clinical endpoint.

The correct mapping is:

Blue line: Combined Model, AUC = 0.912.

Green line: CRP, AUC = 0.784.

Red line: Cystatin C, AUC = 0.847.

The corrected Figures 4, 5 appear below.

**Figure 4:**
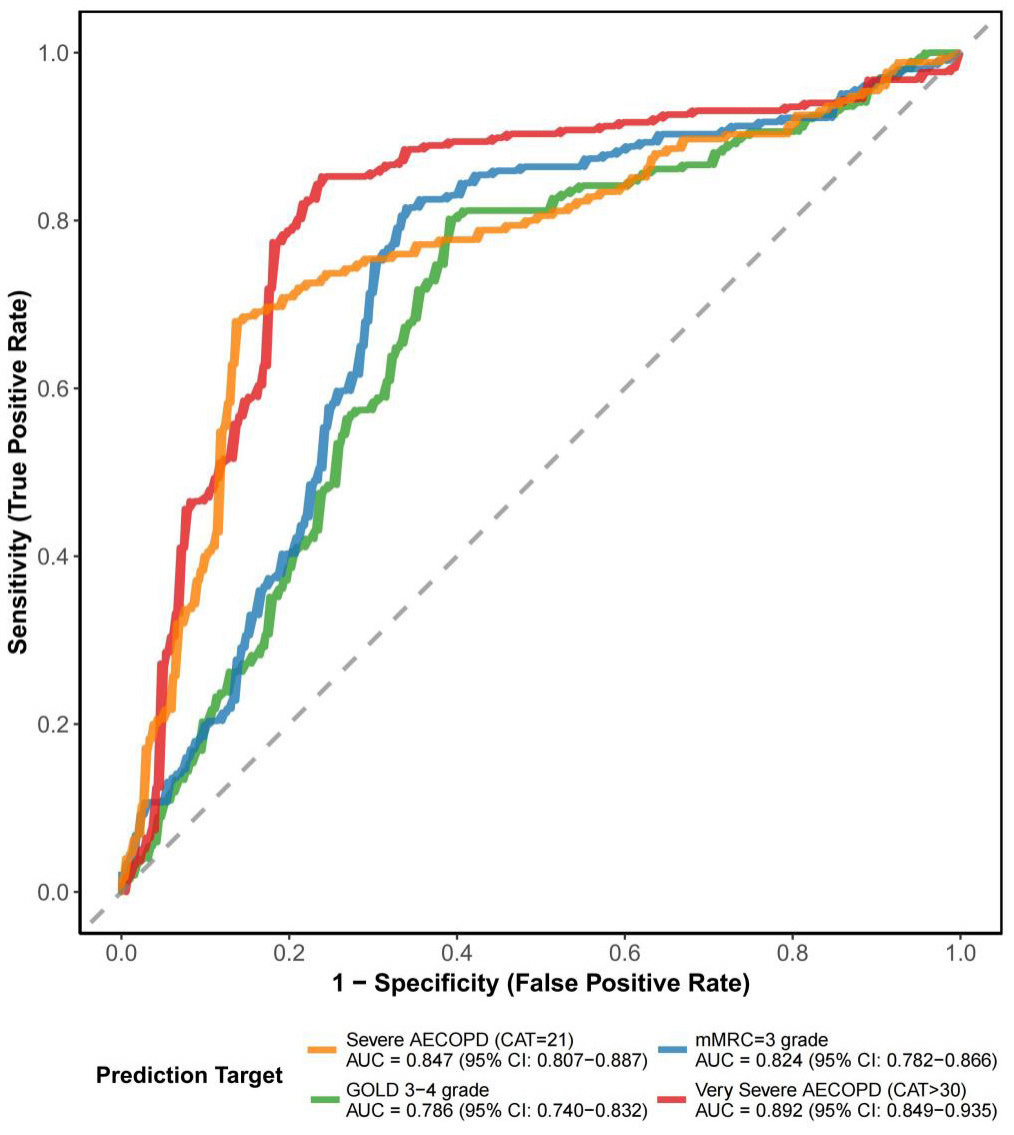
ROC curve for cystatin C in predicting AECOPD severity.

**Figure 5:**
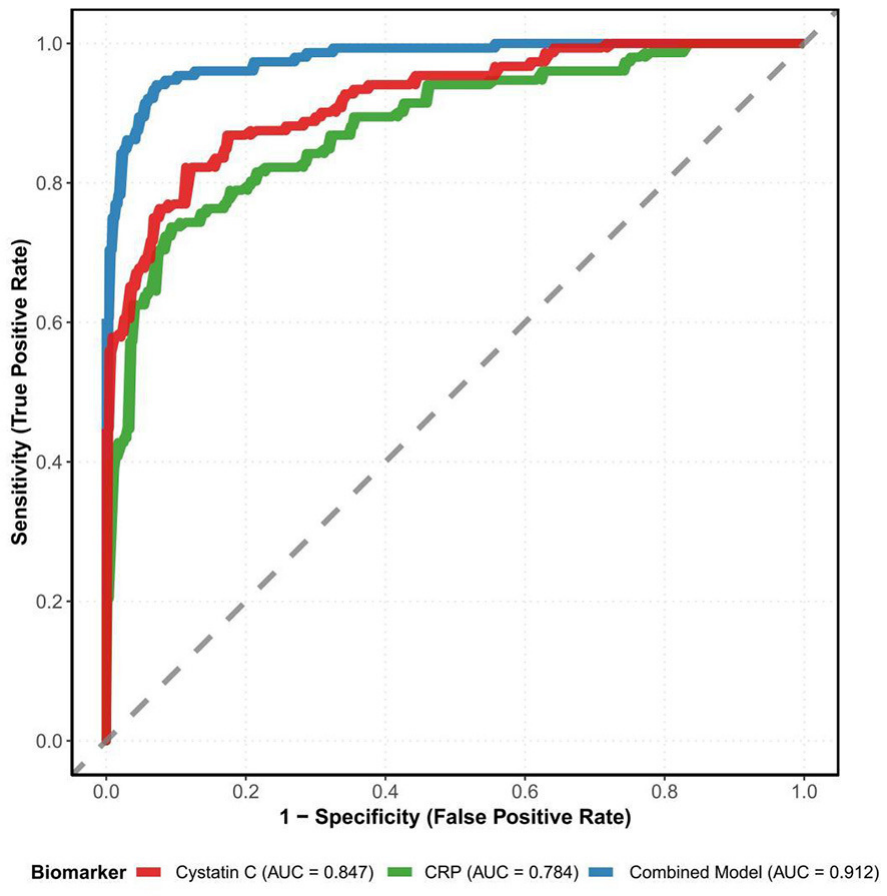
Comparison of ROC curves of different indicators for predicting severe AECOPD.

In the **Abstract**, the case enrollment period was incorrectly stated as “June 2021 to December 2023.” This has been corrected to read:

“The correct study period is “01 January 2024 to 31 December 2025.””

In the Section 2.2 “Study subjects,” the case enrollment period was incorrectly stated as “June 2021 to December 2023.”

A correction has been made to the section, Section 2.2 “Study subjects.”

“The correct study period is “01 January 2024 to 31 December 2025.””

The original version of this article has been updated.

